# A Novel Approach for Treating Lipomas: Percutaneous Microwave Ablation Combined with Liposuction

**DOI:** 10.1007/s00266-024-04091-1

**Published:** 2024-05-09

**Authors:** Shuxun Chen, Yinrong Qiu, Li Lin, Jianhuang Lin, Yizhuo Lu

**Affiliations:** 1 The School of Clinical Medicine, Fujian Medical University, No. 1 Xuefu North Road, Fuzhou, 350122 Fujian China; 2 Department of General Surgery, Zhongshan Hospital of Xiamen University, School of Medicine, Xiamen University, No. 201-209 Hubin South Road, Xiamen, 361004 Fujian China

**Keywords:** Microwave ablation, Liposuction, Lipoma, Cosmetic

## Abstract

Lipomas, benign adipose tissue tumors, are a common occurrence but currently, the options for their treatment are limited, with surgical excision being the most frequently used management pathway. This scenario can often lead to unsatisfactory cosmetic results and significant patient discomfort. This paper introduces a novel technique, percutaneous microwave ablation with liposuction, to address these challenges. The innovative procedure aims to enhance patient satisfaction, minimize post-operative discomfort, and improve aesthetic outcomes. The technique involves two key steps: (1) the application of percutaneous microwave ablation to selectively disrupt the lipoma cells, followed by (2) a targeted liposuction procedure to remove the ablated lipoma tissue. Our approach optimizes the removal of the lipoma and preserves the surrounding healthy tissue, reducing the risk of local recurrence and improving the cosmetic result. The use of preoperative ultrasound imaging allows for precise localization and delineation of the lipoma, aiding in the planning and execution of the procedure. This novel approach to lipoma treatment is reliable, associated with minimal morbidity, and consistently yields effective results. Additionally, it provides a new perspective on lipoma management, potentially changing the paradigm of current treatment approaches.

*Level of Evidence IV* This journal requires that authors assign a level of evidence to each article. For a full description of these Evidence-Based Medicine ratings, please refer to the Table of Contents or the online Instructions to Authors www.springer.com/00266.

## Introduction

Lipoma is the most common benign tumor in clinical practice, originating from adipose cells and often presenting with no apparent symptoms. The incidence rate of lipoma ranges from approximately 1 to 3% [1, 2]. Most lipomas grow in subcutaneous tissues, commonly found in the abdomen, back, neck, and limbs [3]. Various factors are believed to contribute to the development of lipomas, including obesity, unhealthy lifestyle, genetic factors, compression of soft tissues, radiation exposure, and certain medication usage [4]. While lipomas themselves are not malignant, they can have significant negative impacts on patients’ psychological well-being if they grow on the face or in areas that may affect their appearance, if they exert pressure on surrounding tissues or organs due to their large size, or if they form encapsulated structures in critical locations [5]. Currently, surgical excision is the main treatment for lipomas [6]. However, it still carries certain risks, such as scar hypertrophy, infection, and bleeding, which can affect patients’ quality of life to some extent [7].

Microwave ablation, a minimally invasive treatment method, utilizes microwave energy to generate high temperatures and destroy tumors and other abnormal tissues. The principle of microwave ablation involves using a microwave generator to produce ultra-short electromagnetic waves at frequencies of 915 or 2450 MHz and delivering the microwave energy into the tumor tissue via an electrode needle [8]. Under the influence of the microwave field, polar molecules (such as water molecules) within the tumor tissue rapidly move and generate heat through friction, thereby inducing coagulative necrosis in cells and ultimately disrupting the lesion.

Liposuction was first introduced in 1975 and has become a common surgical procedure [9]. Recently, liposuction has been employed for the operative management of lipomas [2]. It has several indications, such as lipodystrophy, gynecomastia, axillary hyperhidrosis, body asymmetry, and lipomas [10].

Therefore, to achieve optimal aesthetic outcomes, we propose a novel approach of percutaneous microwave ablation-assisted liposuction under ultrasound guidance for the treatment of certain head and neck lipomas.

## Methods

This study was designed as a non-randomized controlled trial comparing the outcomes of conventional lipoma excision surgery in 29 patients with those of 17 patients treated using a novel approach of percutaneous microwave ablation combined with liposuction for lipoma treatment. Ethical oversight was ensured through approval by Ethics Committee of Zhongshan Hospital Affiliated to Xiamen University, affirming our commitment to uphold the principles of ethical research practice. Prior to treatment, informed consent was obtained from all participants, ensuring they were fully informed about the study’s purposes, the nature of the procedures involved, and their rights to data protection and voluntary participation. The inclusion criteria were as follows: (1) age over 18 years; (2) presence of a suspected lipoma based on color ultrasound examination; (3) absence of any underlying comorbidities, including known chronic diseases, cardiac conditions, and skin diseases in the treatment area; (4) no use of non-steroidal anti-inflammatory drugs, anticoagulants, platelet inhibitors, immunosuppressants, or similar medications within 2 months prior to enrollment.

A total of 46 patients were included in the follow-up. Among them, 17 patients underwent percutaneous microwave ablation-assisted liposuction for lipoma, while the remaining 29 patients underwent traditional surgical excision. The patients undergoing traditional surgical excision of lipoma were assigned to Group 1, while those receiving percutaneous microwave ablation-assisted liposuction were assigned to Group 2.

## Surgical Techniques

The procedure for percutaneous ablation combined with liposuction is as follows. The microwave treatment system used was produced by Kangyou Medical Technology Co., Ltd. in Nanjing, with the microwave ablation device mainframe model KY-2000A and a maximum microwave output power of 100 W. The microwave ablation needle model was KY-2450A-1, with a working frequency of 2450 MHz. The ultrasound diagnostic instrument was a portable color Doppler ultrasound probe system model M55, from Shenzhen Mindray Bio-Medical Electronics Co., Ltd., equipped with a high-frequency linear array probe L11-3U and a probe frequency of 3–11 MHz.

Before, during, and after the ablation, two-dimensional ultrasound and color Doppler ultrasound examinations were performed on the target lesion. Preoperatively, the lipoma was located using the ultrasound diagnostic instrument, the location and size of the lesion were determined and marked [11]. Color Doppler ultrasound was used to observe the vascular distribution around the lesion, and important large blood vessels were located and marked to avoid injury during surgery [12]. During the operation, the patient was placed in the supine position, with full exposure of the lipoma. The puncture site was routinely disinfected and draped, and 2% lidocaine was used for local anesthesia of the puncture site and path. After the local anesthesia took effect, physiological saline was injected around the lesion to form an isolation zone, separating the tumor from surrounding tissue and preventing thermal injury during the ablation process. The width of the physiological saline interstitial fluid was greater than 1 cm.

**Fig. 1:**
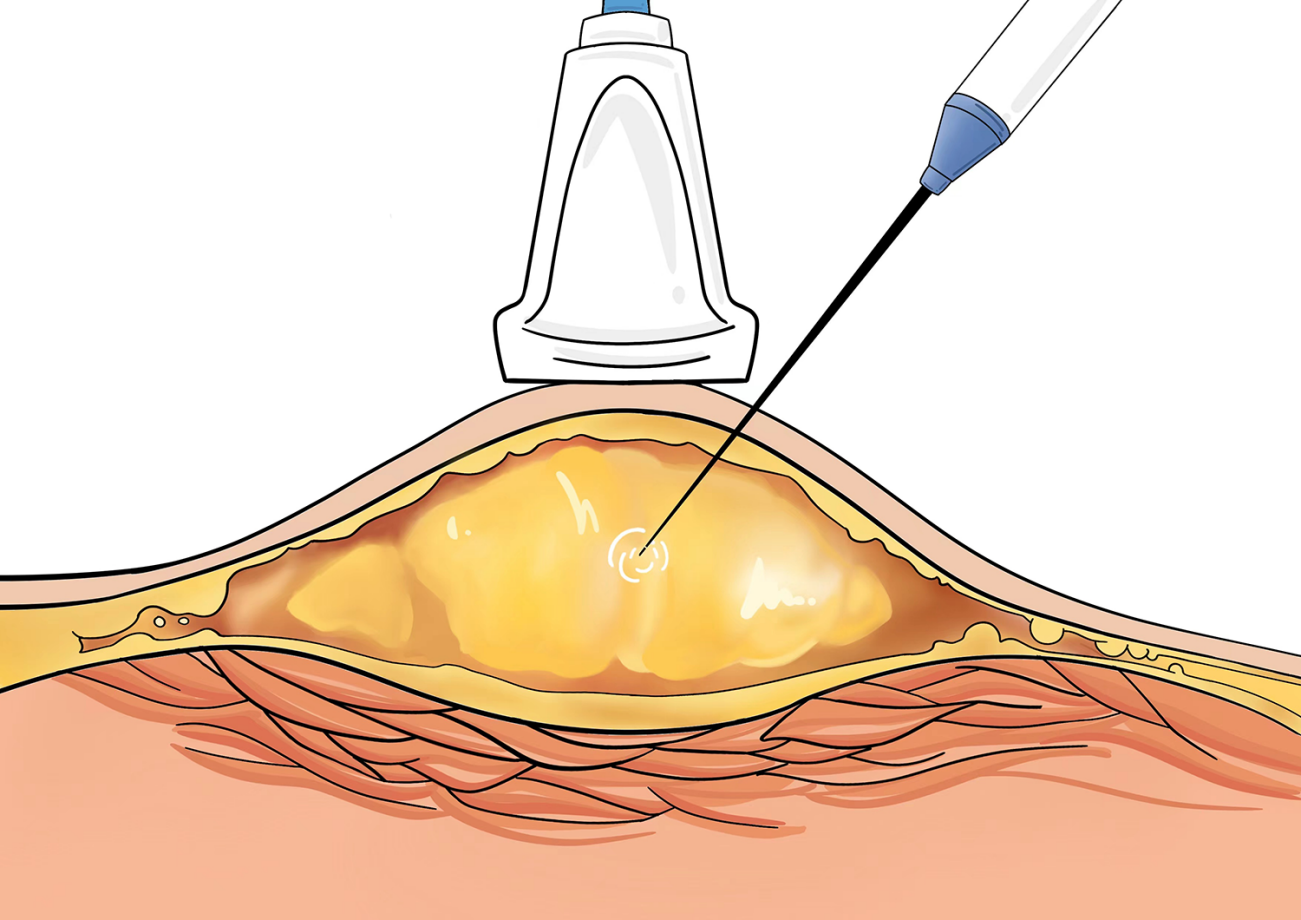
The diagram above shows the process of percutaneous microwave ablation of lipoma

**Fig. 2:**
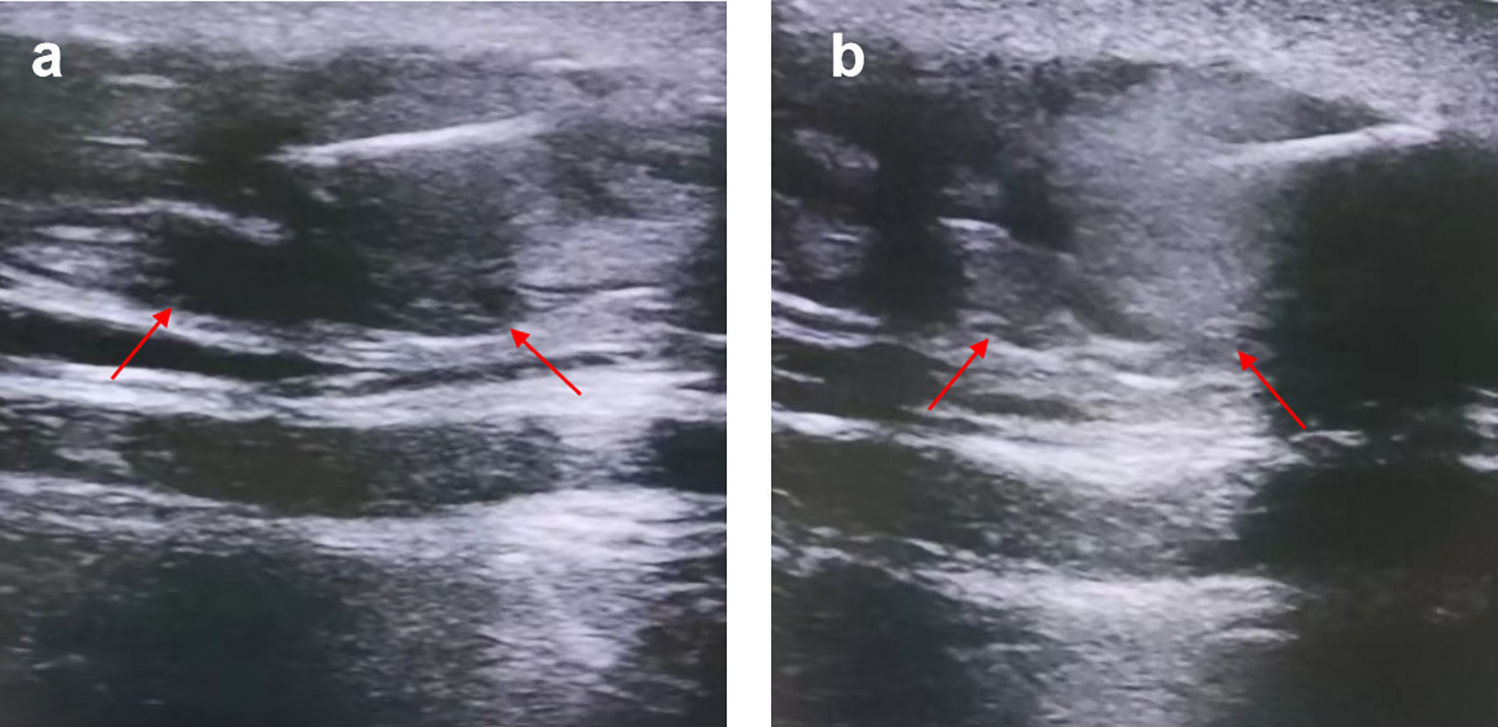
An intraoperative ultrasound image of lipoma ablation; (left) the red arrow shows a low-density lipoma before ablation; (right) the red arrow shows that the density of lipoma after ablation is higher than before

**Fig. 3:**
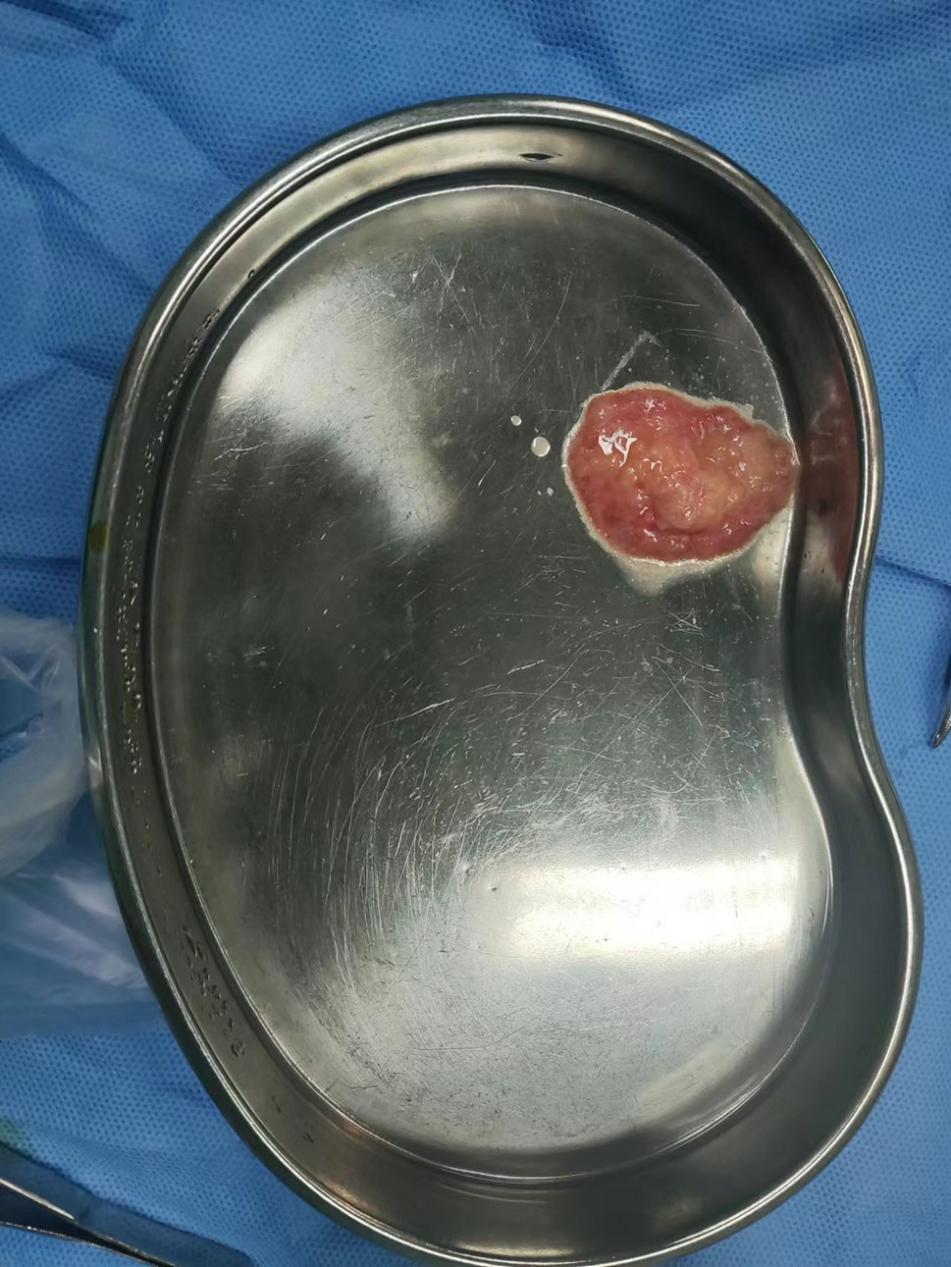
Liquid lipoma tissue aspirated after ablation

Color Doppler ultrasound was used to observe the vascular distribution of the lesion, and under ultrasound guidance, the microwave ablation electrode needle was percutaneously punctured into the lesion. The microwave ablation device was activated for ablation at a power of 15 W. The ablation range of the needle was monitored under ultrasound to avoid damage to normal tissue. Using the microwave ablation needle, multi-point, multi-line, and multi-plane ablations were performed, avoiding vessels under ultrasound guidance (Fig. 1). During the ablation process, it is observable under ultrasound that the lipoma transitions from a low-density shadow to a high-density shadow (Fig. 2). The needle was withdrawn after color Doppler ultrasound showed no blood flow signals. A syringe is used to inhale saline and epinephrine and inject it into the lipoma. The use of adrenaline serves to induce local vasoconstriction, minimizing bleeding and improving the safety and efficacy of the procedure. After 5 min, a blunt liposuction needle with negative pressure was used to suction out the adipose tissue and the ablated tissue (Fig. 3). During needle insertion, the side holes of the liposuction needle faced both sides, and the nature of the aspirated mixture was observed. During suction, a skin fat layer of at least 5 mm was retained, and the remaining adipose tissue was continuously assessed to avoid unevenness caused by excessive suction.

After the lipoma had been sufficiently aspirated, a gauze or pad was used to roll and compress the suction incision, expelling residual swelling fluid, pale red exudate, fragmented fibrous tissue, and adipose tissue. After confirming the absence of active bleeding, routine local disinfection was performed at the needle insertion site, followed by local compression for 5 min. Surgical time, intraoperative blood loss, and other indicators were observed and recorded. Postoperatively, local cold compresses were applied to the treatment area for 6 h.

All patients were discharged on the 2nd day after surgery. All patients returned to our hospital for follow-up at 3 months postoperatively. The surgical time and the general condition of the patients on the 2nd day after surgery were recorded. Patients rated their pain using the NRS score. At the 3-month follow-up, patients’ satisfaction and related complications were recorded. The Global Aesthetic Improvement Scale Scores were used to determine patients’ satisfaction (Table 1). Standardized digital images were obtained, and patients were asked by the research staff to rate their level of overall outcome employing ranging from very satisfied to very unsatisfied.

**Table 1:** The 7-point, descriptive, blinded evaluator-assessed GAIS

Score	Rating	Description
3	Very much improved	Optimal cosmetic result for procedure in this patient
2	Much improved	Marked improvement in appearance from initial condition but not completely optimal for this patient
1	Improved	Obvious improvement in appearance from initial condition
0	No change	Appearance essentially the same as original condition
− 1	Worse	Appearance worse than original condition
− 2	Much worse	Marked worsening in appearance from initial condition
− 3	Very much worse	Obvious worsening in appearance from initial condition

GAIS, Global aesthetic improvement scale

The Numerical Pain Rating Scale were expressed as mean ± standard deviation and compared using the paired-sample t test with SPSS 26.0 software (IBM SPSS Inc., Chicago, IL, USA). A *P* value of < 0.05 was deemed as statistically significant.

## Results

The demographic characteristics of patients undergoing percutaneous microwave ablation combined with liposuction for lipomas are shown in Table 2. The results of follow-up observation showed that 12 patients experienced pain at the treatment site in the initial stage after treatment. This pain gradually alleviated within 2–6 days after the operation. Additionally, we observed mild edema in the local skin of 13 patients shortly after treatment. This edema gradually eased about 1 week after the operation and completely disappeared thereafter. No complications such as fever, wound infection, skin necrosis were observed in all participants. All adverse reactions of patients have been documented (Table 3).

**Table 2:** Demographics of the second group of patients

Sex	No. (%)
Women	13 (82.4)
Man	3 (17.6)
Age category (y)	
10–19	0 (0)
20–29	4 (23.5)
30–39	5 (29.4)
40–49	5 (29.4)
50–59	2 (11.7)
60–69	1 (5.8)
Race	
Asian	17 (100)
Lesion site	
Face	7 (41.1)
Scalp	1 (5.8)
Neck	9 (52.9)
Lipomas grow in other sites	
Torso	4(23.5)
Limbs	4 (23.5)
None	9 (52.9)
The family history of lipoma	3 (17.6)
BMI	
> 24.9	6 (47.1)
18.5-24.9	9 (52.9)
< 18.5	0 (0)

**Table 3:** Statistics of adverse events after percutaneous microwave ablation combined with liposuction

Adverse event	No. (%)
Edema	13 (76.5)
Hematoma	4 (23.5)
Pigmentation	2 (11.7)
Skin burn	0 (0)
Pain/tenderness	10 (58.8)

In this study, we used percutaneous microwave ablation liposuction to treat facial lipomas and observed operation time, safety (bleeding/thromboembolism), treatment effect (tumor size, volume), postoperative recovery (pain at the operation site), imaging performance (color ultrasound), patient satisfaction, etc., to summarize our preliminary experience. The operation time of percutaneous microwave ablation liposuction ranged from 43 to 93 min, with an average of (55.3 ± 16.4) min. No serious complications such as bleeding or thromboembolism occurred after operation in all patients. Only mild local reactions such as skin redness, edema, and bruising occurred, which healed by themselves within a week without intervention.

We carried out B-ultrasound examination of the tumor size of the patients before and after the operation to evaluate the ablation effect of lipoma. This study included 17 patients with facial lipomas, including 3 males and 14 females, ranging in age from 21 to 65 years, with an average age of 36.5 years. Before the operation, the color ultrasound showed that there were round or oval hypoechoic areas in the patients’ facial area, with clear boundaries and uneven echo inside; the average maximum diameter of the tumor was 5.7 ± 1.2 cm (range 3.4–10.3 cm). One week after the operation, the color ultrasound showed no obvious irregular hypoechoic area in the original tumor area, the boundary was blurred, the internal echo was uniform, and no obvious enhancement or blood flow signal was seen. Three months after the operation, the color ultrasound showed no obvious high or low echo area in the original tumor area. There is no evidence of lipoma recurrence or residual signs. In the present study, comprehensive pathological examinations were performed on all resected tissues from the participants. The pathological outcomes consistently corroborated the diagnosis of lipoma, without any indications of liposarcoma or other malignant transformations. Microscopic analysis of the surgical specimens revealed varying degrees of thermal effect on adipose cells that were attributed to the ablation procedure. A proportion of adipocytes exhibited signs of thermal damage, such as cell shrinkage, morphological irregularity, rupture of cell membranes, disintegration of organelles, and necrotic debris. These findings suggest the occurrence of cellular damage in response to the microwave ablation. Conversely, another set of adipocytes retained the characteristic features of benign lipoma, including uniform mature adipocyte size and presence of lipid droplets. This mixture of cellular changes, coupled with clinical data, substantiates the effective targeted destruction of lipomatous tissues by microwave ablation therapy, reaffirming its therapeutic impact.

**Fig. 4:**
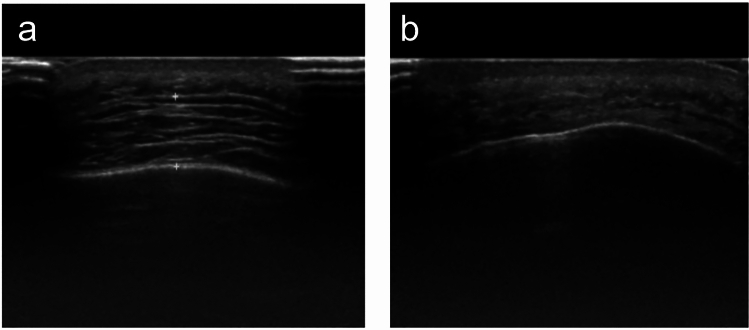
The images above show the ultrasound scans of a 45-year-old male patient with a lipoma on the forehead, before surgery (left) and 3 months post-surgery (right)

**Fig. 5:**
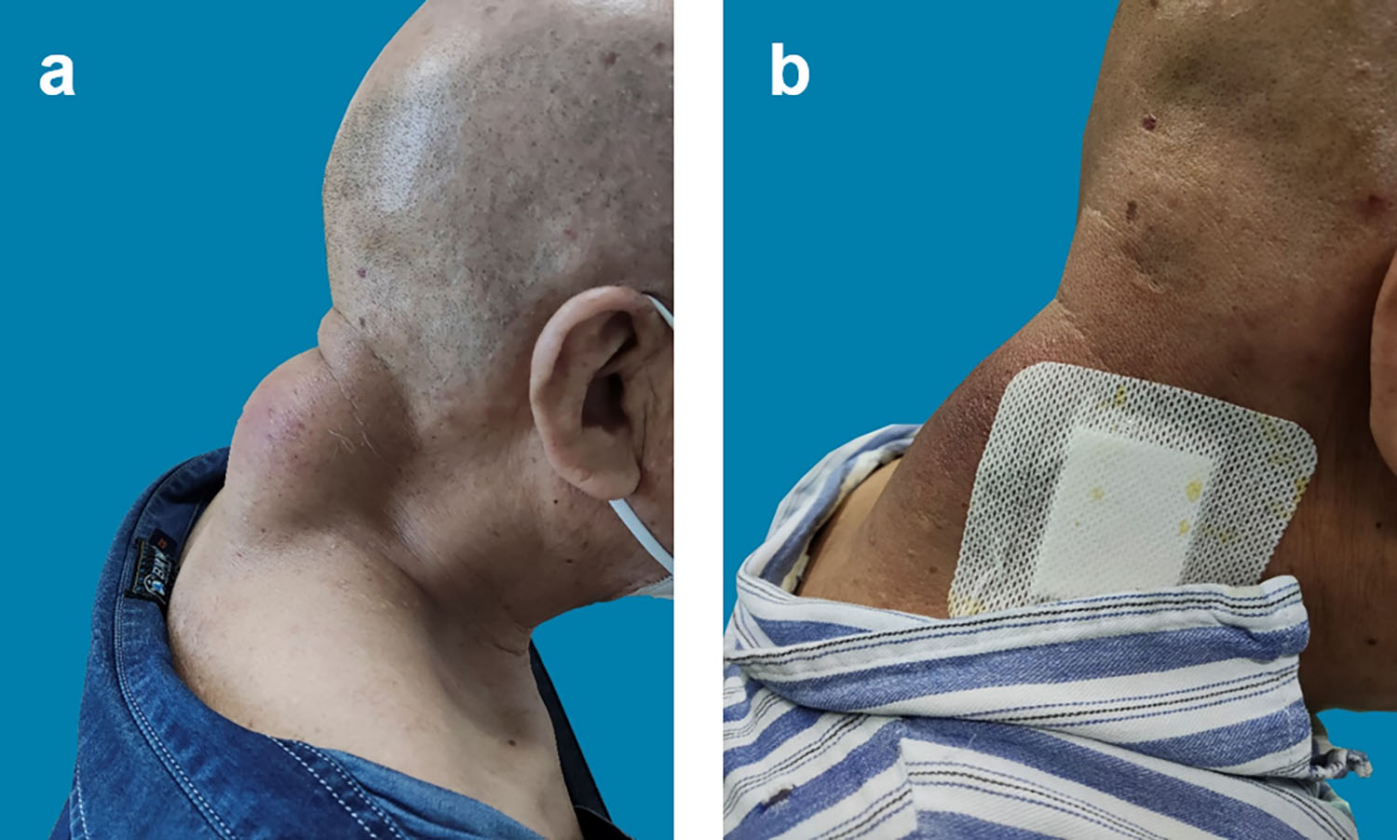
Side view of a 69-year-old patient with posterior cervical lipoma undergoing percutaneous microwave ablation combined with liposuction; (left) side view before receiving treatment; (right) side view just after the surgery

**Fig. 6:**
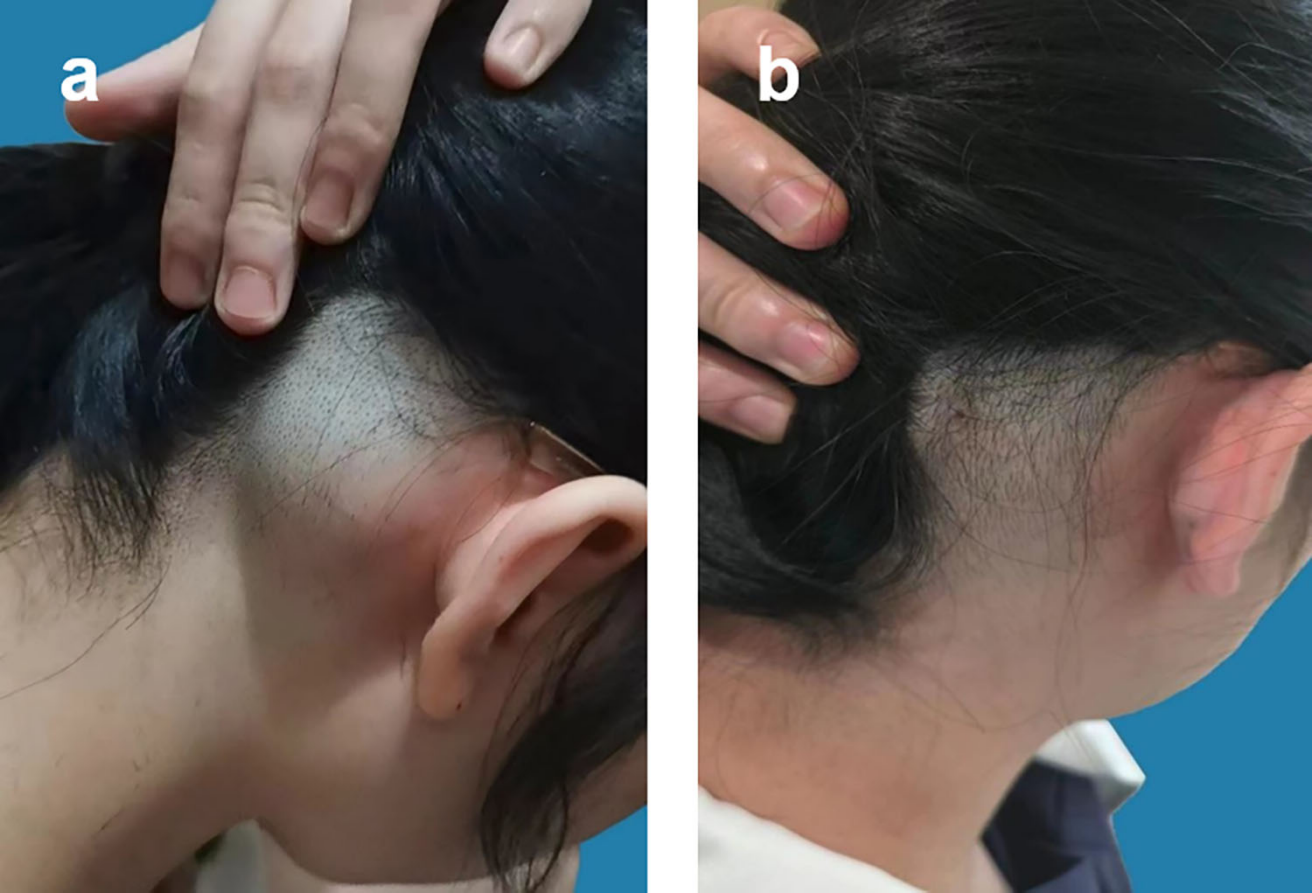
A side view of a 23-year-old woman with right occipital lipoma undergoing percutaneous microwave ablation combined with liposuction; (left) side view before receiving treatment; (right) side view 1 week after the surgery

**Fig. 7:**
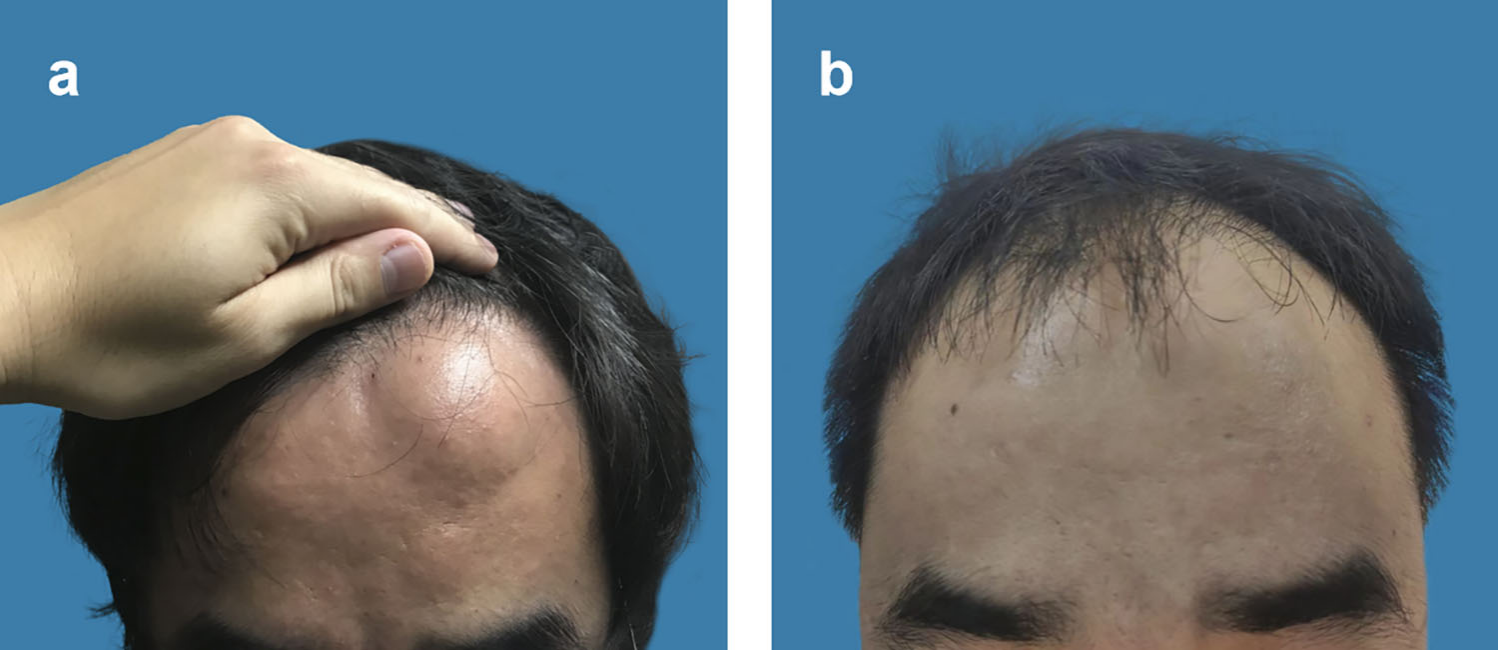
Frontal view of a 39-year-old male with left frontal lipoma undergoing percutaneous microwave ablation combined with liposuction; (left) frontal view before receiving treatment; (right) frontal view 3 month after the surgery

The patients included in this study underwent pain and functional assessments within 1 day and 1 week after surgery. The Numerical Pain Rating Scale (NPRS) was used to evaluate the patients’ pain levels, ranging from 0 (no pain) to 10 (severe pain). For the first group of patients, those in the traditional surgery group, the NPRS score on the 1st day after surgery was 4.3 ± 0.7, and the score 1 week post-surgery was 0.6 ± 0.8. For the second group of patients, those in the percutaneous ablation group, the average NPRS score on the 1st day after surgery was 3.2 ± 0.8, and 1 week post-surgery, the average NPRS score was 0.3 ± 0.1. The recovery of skin sensation and mobility in the surgical site was normal, without any apparent abnormalities. The satisfaction level of patients who underwent percutaneous microwave ablation combined with liposuction surgery 1 week after the operation was recorded (Table 4). Follow-up questionnaires were conducted on the patients at postoperative 90 days (Table 5). The ultrasound images of the patient before surgery and 90 days postoperatively are shown below (Fig. 4). The patient’s appearance before and 90 days after surgery is shown in the following figure (Figs. 5a and b, 6a and b, 7a and b). The data of Blinded Evaluator-Assessed GAIS 1 month and 3 months after surgery are shown below (Table 6).

**Table 4:** Patient satisfaction

Rating (*n* = 17)	No. (%)
Very satisfied	5 (29.4)
Satisfied	8 (47.0)
Somewhat satisfied	3 (17.6)
Somewhat unsatisfied	1 (5.8)
Unsatisfied	0 (0)
Very unsatisfied	0 (0)

**Table 5:** Patient questionnaire

Questionnaire	Score
Are there any difference in color between the treated area and the normal skin?	1.05 ± 0.22
Are there any stretching or widening at the edges of your treatment site?	1.00
Are there any sensations of itching in the treated area?	1.11 ± 0.31
Is the surface of the treated area irregular?	1.11 ± 0.31
Is there a perceived increase in hardness of the treated area?	1.05 ± 0.22
Is the treated area more prone to breakage compared to the untreated area?	1.00
Does the treated area experience a sensation of tightness during activities?	1.00
Does the treated area experience any allergic reactions?	1.00

^*^5-point scale:1 indicates close to normal; 5 indicates highly abnormal

**Table 6:** Blinded evaluator-assessed GAIS

	Traditional group		Ablation group	
	Self-assessment	Peer assessment	Self-assessment	Peer assessment
1 month	− 0.14	0.24	0.71	1.52
3 month	0.79	− 0.04	1.88	2.23

## Discussion

Lipomas are benign soft tissue tumors composed of mature adipocytes and are among the most common mesenchymal tumors [13]. However, for lipomas located in areas such as the head and neck that cannot be covered by clothing, exploring alternative approaches to traditional surgical excision becomes crucial. Initially, liposuction was proposed as an alternative to conventional surgery [14]. This method has been proven effective in removing lipomas with a diameter exceeding 4 centimeters, as well as adjacent multiple lipomas [15]. The liposuction technique requires only one incision site, significantly reducing the number of postoperative scars. However, the efficacy of this method in removing small lipomas is limited, leading to its limited application [16].

Another study on non-invasive removal of lipomas involved the use of lipostabil, which is based on phosphatidylcholine [17–19]. However, injecting excessive doses of the lipolytic agent into poorly perfused or scarred tissues, or injecting it at incorrect anatomical levels, can potentially lead to serious adverse reactions, such as hematoma, muscle damage, nerve injury, skin ulceration, necrosis, or blistering [20].

The ablation has been widely applied in the treatment of benign nodules in the thyroid, liver, and lungs. In 2018, Ji Yeon Hong successfully demonstrated the reduction of facial lipomas using radiofrequency ablation [21]. However, tissue aspiration was not performed after ablation, which strictly speaking, carries a significant risk of recurrence. In the context of our study on lipoma surgery, the strategic modulation of microwave emission—both in terms of power and duration—according to the specific dimensions and positioning of the tumor is paramount. This ensures effective ablation outcomes with minimal impact on adjacent healthy tissues. Additionally, the adoption of pulsed rather than continuous microwave emission significantly reduces the thermal load on surrounding tissues. This approach effectively decreases the risk of thermal damage to sensitive areas. Moreover, whenever feasible, we strongly recommend the intraoperative employment of nerve monitoring technologies. These devices enable the real-time identification and preservation of vital neural structures in the vicinity of the lipoma, adding an extra layer of safety to the ablation process. Collectively, these carefully considered measures bolster the precision and safety of MWA, striking a delicate balance between achieving therapeutic efficacy and preserving critical anatomical features.

The combination of percutaneous microwave ablation guided by ultrasound and liposuction may have several advantages. Firstly, this procedure is performed under direct ultrasound visualization, allowing the operator to avoid important blood vessels or muscle tissues [22]. Secondly, the use of microwave ablation can liquefy the lipoma, facilitating the removal of adipose tissue during liposuction. The combination of these two techniques maximizes the excision of the lipoma, avoiding the severe inflammatory reactions or skin necrosis associated with residual fat-dissolving agents, such as phosphatidylcholine. Additionally, this approach, which utilizes incisions to eliminate the lipoma, minimizes the need for postoperative dressing changes.

Postoperative edema is the most common complication of this procedure and needs to be thoroughly explained and clarified to patients. This may be due to the injection of a sufficient amount of physiological saline solution into the ablation zone during the procedure. On one hand, the physiological saline solution aids in separating the tumor mass from the surrounding normal tissues before ablation; on the other hand, it dilutes the liquefied fat resulting from microwave ablation. These factors, combined with the fluid exudation from the wound itself, inevitably lead to postoperative edema at the primary tumor site. Approximately 93% of the patients who undergo percutaneous microwave ablation combined with liposuction experience varying degrees of postoperative edema. Its manifestation ranged from mild to moderate in the majority of patients and resolved spontaneously within the 1st week post-surgery. We categorized the observed edema into three distinct subclasses based on severity: mild (minimal swelling, no functional impairment, resolved within 3–5 days), moderate (noticeable swelling with some discomfort, resolved within 7 days), and severe (pronounced swelling leading to functional impairment or requiring medical intervention). Our data revealed that the vast majority of patients experienced mild to moderate edema (88%), which resolved without medical intervention. 5% experienced severe edema, which was managed effectively with therapeutic measures such as compression therapy and, in some cases, the use of diuretics. The transient nature of edema and its predictable resolution trajectory suggests that while it is the most common complication, it generally does not result in long-term adverse outcomes for patients undergoing percutaneous microwave ablation combined with liposuction for lipoma treatment. The higher incidence of this complication necessitates a pragmatic discussion with patients during the informed consent process, setting realistic expectations about the recovery period and the likelihood of temporary swelling post-procedure.

Before injection, clinicians need to exclude malignant connective tissue tumors and other possible causes of soft tissue masses. These reasons may include liposarcoma, lymphoma, osteosarcoma, cutaneous metastatic malignancies, as well as epidermoid cysts, foreign body granulomas, rheumatoid nodules, gouty stones, etc. To exclude malignant tumors, a diagnosis of lipoma must be made and sufficient data collected before liposuction. Atypical lipomas or liposarcomas may have a similar appearance, which should always be kept in mind. Diagnostic methods such as fine-needle aspiration and ultrasound imaging can be helpful. These examinations can assist doctors in determining the nature and origin of the mass, thus formulating further treatment plans. Therefore, performing these examinations before injection is important to ensure patient safety and treatment accuracy. Although our series did not encounter any cases ultimately diagnosed as liposarcoma in the postoperative pathological examination, we uphold the belief that for patients whose postoperative pathology confirms liposarcoma, an extended radical surgery is imperative [23]. This involves the surgical removal of surrounding tissues to eliminate any remaining malignant cells and reduce the risk of recurrence. Should it be necessary, the treatment plan may also incorporate chemotherapy or radiation therapy to further ensure the thorough eradication of malignant cells and to minimize the potential for recurrence [24].

None of the patients in this study experienced severe complications; however, percutaneous microwave ablation combined with liposuction still carries potential risks, such as bleeding, fat embolism, uneven liposuction area, postoperative chronic pain, hematoma, wound infection, nerve damage, and pigment changes. During postoperative follow-up, some patients with loose skin could feel the mass in the original treatment area which was different from the surrounding tissue texture, and a few patients could still see a slight protrusion in the original treatment area. This condition usually disappears within 3 months after the operation, so we speculate it may be a postoperative hematoma [25].

The management of lipomas has traditionally relied on surgical excision, which, despite its efficacy, possesses several limitations, including potential for scarring, pain, and a longer recovery period. In contrast, our novel technique—percutaneous microwave ablation combined with liposuction—offers a less invasive alternative, which aligns with the current trend toward minimally invasive procedures in surgical oncology and soft tissue management. Unlike conventional surgical excision, which typically removes the lipoma in its entirety but risks significant scarring and longer recovery, our technique utilizes microwave ablation to selectively heat and destroy lipoma cells. This is followed by liposuction to remove the liquefied tissue, minimizing the surgical footprint and preserving surrounding healthy tissue. This method mitigates the risk of large scars and has shown a lower complication rate, placing it as a competitive alternative to traditional approaches. Studies have reported the safety and effectiveness of microwave ablation in other contexts, such as liver and kidney tumor management, underscoring the potential for its application in lipoma treatment [26, 27]. Modern patient care increasingly prioritizes not only treatment efficacy but also quality of life and cosmetic outcomes. Our technique directly addresses this trend by offering superior cosmetic results with less postoperative pain and quicker recovery times. In our study, patient satisfaction scores were notably higher for those treated with microwave ablation and liposuction compared to historical cohorts undergoing traditional excision, reflecting the importance of cosmetic outcomes in patient-centered care. While the initial cost of microwave ablation equipment may be higher than the tools required for conventional surgical excision, the overall cost savings derived from shorter hospital stays, reduced need for postoperative care, and quicker return to daily activities make our technique economically attractive. Moreover, the reduced risk of complications and subsequent interventions further underscores the cost-effectiveness of our approach.

In conclusion, our technique presents a promising alternative to traditional surgical excision of lipomas, offering advantages in terms of invasiveness, recovery time, cosmetic outcomes, and patient satisfaction. As the medical community continues to embrace minimally invasive procedures, it is paramount that we evaluate not only the clinical efficacy but also the broader impacts on patient quality of life and healthcare economics. Our findings contribute to this evolving narrative, suggesting that percutaneous microwave ablation combined with liposuction could become a new standard for lipoma treatment in the near future.

To determine the optimal parameters for percutaneous microwave ablation combined with liposuction, large-scale controlled studies are still needed. Such studies could involve a larger number of patients and compare the treatment group with a control group, further evaluating the efficacy and advantages of this treatment method, and providing more scientific support for clinical practice.
